# Discontinuity of social support among US adults with cognitive impairment before and after the confirmed diagnosis of dementia: a matched ambidirectional cohort study

**DOI:** 10.1186/s12916-025-04264-y

**Published:** 2025-07-15

**Authors:** Huanyu Zhang, Benjamin R. Underwood, Sabina London, Huitong Zhao, Jiazhou Yu, Da Feng, Shanquan Chen

**Affiliations:** 1 Clinical Big Data Research Center, The Seventh Affiliated Hospital, Sun Yat-Sen University, Zhenyuan Road, Shenzhen, 518107 Guangming District China; 2 Department of Psychiatry, University of Cambridge, 18B Trumpington Road, Cambridge, CB2 0SZ UK; 3 Cambridgeshire and Peterborough NHS Foundation Trust, Cambridge Road, Cambridge, CB21 5EF UK; 4 University of Connecticut School of Medicine, 263 Farmington Avenue, Farmington, CT 06030 USA; 5 Population and Development Research Center, Beijing, China; 6 Department of Medicine and Therapeutics, The Chinese University of Hong Kong, Hong Kong SAR, China; 7 School of Pharmacy, Huazhong University of Science and Technology (HUST), Wuhan, 430030 China; 8 School of Public Health, The University of Hong Kong, Hong Kong, 999077 China; 9 International Centre for Evidence in Disability, London School of Hygiene & Tropical Medicine, Keppel Street, London, WC1E 7HT UK

**Keywords:** Dementia, Social support, Continuity of care, Race/ethnicity, Sex

## Abstract

**Background:**

Despite increased attention on dementia, much remains unknown about the integration of clinical and non-clinical care, particularly regarding long-term social support, a primary source of non-clinical care. This study uniquely examines the effect of receiving a formal dementia diagnosis on the continuity of social support, an understudied transition point in dementia care pathways.

**Methods:**

In this ambidirectional cohort study, we examined ten waves of data from the Health and Retirement Survey(HRS) for US adults over 50 through 2000–2018. Eligibility was limited to participants with cognitive impairment. The exposure group were people with a confirmed dementia diagnosis (*N* = 1261), and the control group were matched by age, sex, race/ethnicity, and survey wave, but without a confirmed diagnosis (*N* = 12,604). Unmet social support was defined as reporting physical disability without receiving corresponding social support. Physical disability was assessed using measures of basic activities of daily living (BADLs) and instrumental activities of daily living (IADLs). The data were fitted using controlled interrupted time series analysis to explore the continuity of unmet social support before and after a diagnosis.

**Results:**

After dementia diagnosis, adults experienced a significant increase in unmet IADL support needs (coef = 0.10, 95% CI [0.07, 0.13]), particularly for making phone calls (coef = 0.74, 95% CI [0.16, 1.33]). By race/ethnicity, Hispanics showed a significant rise in unmet BADL support needs (coef = 0.74, 95% CI [0.03, 1.46]), especially for eating assistance (coef = 1.58, 95% CI [0.17, 2.99]). Blacks experienced increased unmet BADL needs in toileting (coef = 1.52, 95% CI [0.57, 2.47]) and IADL support (coef = 0.09, 95% CI [0.00, 0.17]). Sex disparities were also identified, with females showing decreased unmet BADL support(coef =  − 0.55, 95% CI [− 1.03, − 0.06]) but increased unmet IADL support (coef = 0.08, 95% CI [0.04, 0.11]), while males experienced increased unmet toileting (coef = 0.78, 95% CI [0.03, 1.53]) and IADLs support (coef = 0.14, 95% CI [0.10, 0.18]).

**Conslusions:**

Our study identifies a disconnect in the care provided to individuals with dementia before and after their diagnosis. Notably, post-diagnosis, we observed substantial disparities in unmet social support needs across various racial groups. This highlights the need for more cohesive and equitable care strategies in the dementia care continuum.

**Supplementary Information:**

The online version contains supplementary material available at 10.1186/s12916-025-04264-y.

## Background

Cognitive impairment and dementia involve memory loss and a decrease in cognitive functions which result in growing functional restrictions over time such as disorientation, impaired communication abilities, and diminished judgement in decision-making tasks [1, 2]. This progressive condition brings considerable challenges and burdens not only to the affected individuals but also to their families, healthcare providers, and broader society, highlighting the critical need for ongoing management of their condition [3, 4]. Continuity of care is defined as the seamless and coordinated transition of patients across various healthcare services and providers throughout the course of a chronic disease [5]. For individuals suffering from complex chronic conditions such as dementia, ensuring continuous care is essential for delivering care that is patient-centered, comprehensive, and well-coordinated [5–7]. Research in other chronic conditions, such as diabetes and cardiovascular diseases, has underscored the benefits of continuous care in improving patient outcomes and reducing hospitalizations [8]. The demand for such continuous care among dementia patients is substantial and continues to grow In the United States (U.S.), approximately 6.7 million seniors aged 65 and older are living with Alzheimer’s disease, the most prevalent form of dementia, and around 22% of Americans aged 65 and above are estimated to have mild cognitive impairment [9].

Continuity of care for patients with dementia spans both clinical services such as diagnosis, treatment, and follow-up at hospitals and clinics, as well as long-term non-medical care like home health services, caregiver education and training, and community support programs [5]. The World Alzheimer Report 2009 outlines a seven-stage continuum of care model for dementia that primarily focuses on providing support post-diagnosis and for caregivers once a diagnosis is confirmed [10]. A study conducted in the U.S., involving a sample of 1158 community-dwelling individuals, highlighted the substantial amount of caregiving required over the first eight years after the onset of dementia, with an average of 9 h per day of caregiving [11]. However, evidence suggests suboptimal continuity across these domains. Qualitative studies have highlighted poor communication and coordination between hospital staff and community care providers during care transitions, resulting in lapses in treatment and patient monitoring [12–16].

Previous research on continuous care of people with dementia primarily focuses on services following discharge [17–24]; there has been limited quantitative research into how dementia care and support are maintained following diagnosis. In the U.S., a few studies have looked into care continuity for individuals diagnosed with dementia, particularly during transitions between healthcare facilities [14, 25]; yet research is scarce on transitions from healthcare settings to community care. A study from Taiwan highlighted the negative outcomes of insufficient continuity, showing that people with dementia experiencing larger gaps in outpatient clinic follow-ups had increased emergency room visits and hospitalizations [26]. This underscores, also supported by a literature review [27], a broader need for evidence-based research focused on care pathways and transitions to community settings, aiming to enhance follow-up planning and support for people with dementia more effectively.

Utilizing a matched ambidirectional cohort methodology, where data are analyzed both retrospectively and prospectively, this study draws on data from the Health and Retirement Survey (HRS) to fill the existing evidence gap. It examines the dynamics of unmet social support needs in a longitudinal group of older adults in the U.S., focusing on the period before and after receiving a clinical diagnosis of dementia. This study design allows for a comprehensive analysis of changes over time, both prior to and following the dementia diagnosis, providing a more nuanced understanding of social support needs. Through the application of interrupted time series analysis, we have quantitatively evaluated any immediate changes and long-term patterns in disability-related social support gaps, investigated variations across sociodemographic groups, and explored potential drivers of observed discontinuities in care. Based on principles of effective healthcare delivery, our hypotheses are twofold: firstly, there will be at least no immediate increase in the levels of unmet social support needs following a confirmed diagnosis of dementia; and secondly, the trajectory of unmet social support needs will either decline or remain stable in post-diagnosis. In this study, unmet social support needs specifically refer to instances where individuals have functional disabilities but do not receive corresponding assistance—a key indicator of gaps in the care continuum. Unlike previous research that simply describes increased care needs in dementia, our study specifically examines whether the critical transition point of receiving a formal diagnosis affects the alignment between disability and corresponding social support. This approach allows us to evaluate if the healthcare system effectively leverages diagnosis as an opportunity to mobilize appropriate support services.

## Methods

### Study design and participants

We performed a matched ambidirectional cohort study using ten waves (2000–2018) of data from the HRS, a nationally representative and biennial study of U.S. adults aged 50 years or older. The HRS collects data through interviews (in-person, phone, or online) on sociodemographics, health, and cognitive status, either self-reported or via a proxy if the respondent is unable or unwilling to complete the interview, as described elsewhere [28, 29].

We selected this study period to avoid data affected by the COVID-19 pandemic. The index date was defined as the later of either a participant’s entry into the study or the study start date. Follow-up ended at death, withdrawal, or the study end date.

Participants were eligible if they had potential cognitive impairment or a confirmed diagnosis of dementia in any wave. Cognitive status was assessed using a validated algorithm based on the Telephone Interview for Cognitive Status (TICS) and proxy index [30–33]. The TICS is a 27-point cognitive scale that includes an immediate and delayed 10-noun free recall test, a serial sevens subtraction test, and a backwards-count-from-20 test. The proxy index is a 11-point scale, covering the subject’s memory, limitations in five instrumental activities of daily living (IADLs) (defined below), and difficulty completing the interview because of a cognitive limitation. Potential cognitive impairment was defined as a TICS score ≤ 11 or a proxy index score ≥ 3. Full details about the TICS and proxy assessment can be found elsewhere [30–33]. Diagnosis of dementia was judged by questions “Has a doctor ever told you that you have dementia, senility or any other serious memory impairment?” and “Has a doctor ever told you that you have Alzheimer’s Disease?” with responses yes or no.

**Fig. 1:**
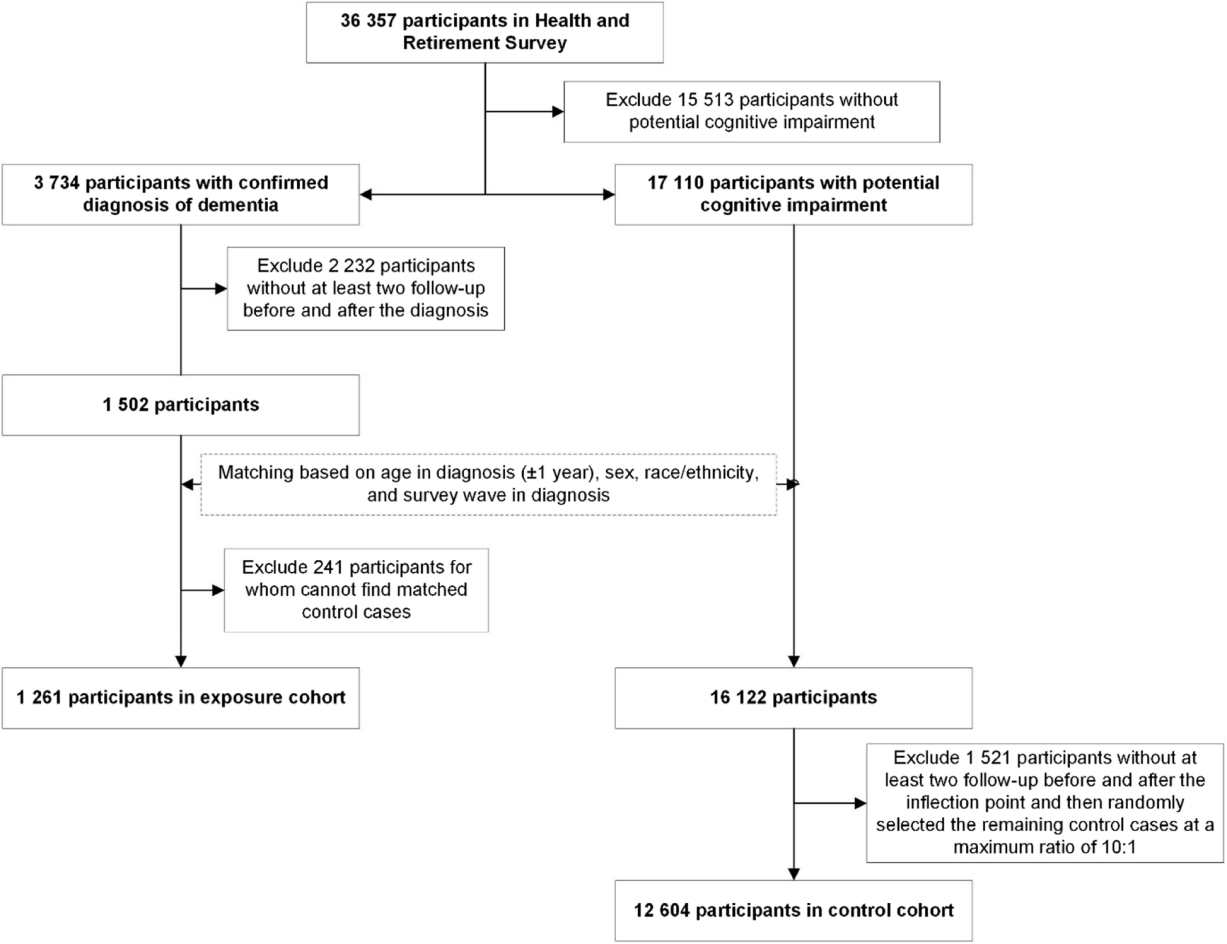
STROBE diagram showing the selection of the participants and matching

The exposure group included participants with a confirmed dementia diagnosis and at least two waves of data before and after diagnosis. The diagnosis wave was defined as the “intervention point”. The control group was drawn from the remaining participants of HRS with potential cognitive impairment but without a dementia diagnosis. Controls were matched to exposure cases based on age at diagnosis (± 1 year), sex, race/ethnicity, and survey wave when diagnosed, and had to have two waves before and after the matched intervention point. Matching was followed by exclusions for inadequate follow-up, and final selection was done at a maximum 10:1 ratio (controls:cases). The procedure is summarized in Fig. 1.

The data are publicly available. The use of secondary de-identified data makes this study exempt from institutional review board review. This study follows the Strengthening the Reporting of Observational Studies in Epidemiology (STROBE) reporting guideline [34].

### Outcome and measures

Unmet social support was defined as reporting physical disability without receiving corresponding social support. This measure captures the mismatch between functional needs and received assistance, rather than a general measure of social support. Physical disability was assessed using 11 items in total: six basic activities of daily living (BADL)—dressing, walking across a room, bathing, eating, getting in and out of bed, and toileting—and five items of instrumental ADLs (IADL)—preparing a hot meal, shopping for groceries, making phone calls, taking medications, and managing money [35–37]. For each disability item, respondents were asked if they had difficulty performing the activity (indicating a disability) and separately, whether they received help from others for that specific activity (indicating social support), including assistance from family, friends, neighbors, or formal caregivers. Based on this, we constructed a total of 16 outcome variables, including 2 continuous outcomes measuring the number of unmet BADL and IADL support needs, and 14 categorical outcomes—comprising two overall indicators of having any unmet BADL or IADL support and twelve binary indicators for unmet support across each of the 11 individual BADL and IADL items. This operationalization is consistent with prior research examining gaps in care and has been used in previous studies of care needs among older adults [38–40].

### Covariates

We examined the sociodemographic variables, including age (in years), sex (female vs male), race/ethnicity (Hispanic, non-Hispanic Black, or non-Hispanic White), living alone (yes or no), number of children, and per capita household income. Due to small sample sizes, individuals identifying as Asian, Native American, Pacific Islander, or other ethnicities were excluded from the final analysis to ensure adequate statistical power and stability of estimates. Income was the sum of household capita income received over the last calendar year, including business or farm income, self-employment earnings, gross rent, dividend and interest income, trust funds or royalties, and other asset income. We also examined whether the respondent was reported by a proxy (yes or no) and covered by long-term care insurance (yes or no).

### Statistical analysis

To describe the characteristics at intervention point, categorical variables were reported as number (percentage), and continuous variables were reported as mean (standard deviation, SD). Differences between groups were assessed via two-tailed t-tests (for continuous variables) and chi-square tests (for categorical variables).

We fitted the data using a controlled interrupted time series (CITS) design by the following model to explore the continuity of unmet social support before and after the confirmed diagnosis. CITS, a quasi-experimental approach, is used to evaluate the effectiveness of an intervention or treatment by tracking over an extended period both before and following the intervention [41].

1 $$y = \beta 0 + \beta 1\cdot \text{Phase}+ \beta 2\cdot \text{Wave }+ \beta 3\cdot \text{Phase}\cdot \text{Wave }+ \beta 4\cdot \text{Exposure }+ \beta 5\cdot \text{Exposure}\cdot \text{Wave }+ \beta 6\cdot \text{Exposure}\cdot \text{Phase }+ \beta 7\cdot \text{Exposure}\cdot \text{Phase}\cdot \text{Wave }+\text{ Covariates}+\upvarepsilon +\upgamma +\updelta$$

where *y* is the outcome of interest; Phase indicates “before/after diagnosis” (0 before diagnosis or the equivalent period in control cases, and 1 after diagnosis or the equivalent period in control cases); Wave is the wave point of data (within a phase, − *n*… − 2, − 1, 0[intervention point], 1, 2, …, *n*); Exposure indicates confirmed diagnosis (0 for control cohort, or 1 for exposure cohort); Covariates included age, sex or race/ethnicity, per capita household income, proxy status, long-term care insurance, and survey year in intervention point; $\varepsilon$ is residual in individual level; $\gamma$ is residual in follow-up level; $\delta$ is residual in matched cluster level.

The coefficients in Eq. 1 are described elsewhere [41]. Two coefficients are of particular importance concerning diagnosis. β6 reflects the step change (instantaneous effect) resulting from confirmed diagnosis, over and above any equivalent change that may have occurred in the control cohort. β7 represents the longer-term or trend effect of confirmed diagnosis, namely the slope change in interested outcome following diagnosis (after any instantaneous effect and relative to the pre-diagnosis slope), over and above any equivalent change in the control cohort.

Equation 1 was fitted by multilevel linear regression for continuous outcomes and multi-level logistic regression for binary outcomes.

We conducted one sensitivity analysis by matching the control cohort based on all general people.

All analyses were completed using R, version 4.3.0. We report two-tailed *p* values and 95% confidence intervals (CIs) throughout. *P* < 0.05 was considered to be statistically significant.

## Results

### Basic description

Between 2000 and 2018, a total of 1261 individuals with diagnosed dementia were included in the exposure cohort, and 12,604 individuals with potential cognitive impairment were included in the control cohort (Fig. 1). There were some differences in the characteristics of patients between the two cohorts at the intervention point (wave of diagnosis) (Table 1). Individuals in the confirmed diagnosis cohort were more likely to be living alone (*p* = 0.008) and to be proxy-reported (*p* < 0.001). The diagnosed group showed significantly lower cognitive performance on the telephone interview for cognitive status (6.19 vs 12.47, *p* < 0.001) and higher scores on proxy-assessment of cognitive impairment (4.15 vs 2.45, *p* < 0.001), confirming greater cognitive impairment in the diagnosed group. No significant difference between the two cohorts was observed for other socio-demographic characteristics, including age (*p* = 0.673), sex (*p* = 1.000), race/ethnicity (*p* = 0.999), per capita household income (p = 0.319), having long-term care insurance (*p* = 0.281), and number of children (*p* = 0.657). Compared to people in the control cohort, individuals in the confirmed diagnosis cohort were more likely to have physical disability (*p* < 0.001), but also more likely to be the receipt of corresponding social support (*p* < 0.001).

**Table 1:** Basic description of the sample at the quasi-intervention or intervention point. Data are shown as mean (SD) for continuous variables and number (percentage) for categorical variables. *P* values were extracted from two-tailed *t*-tests (for continuous variables) and chi-square tests (for categorical variables). *BADL* basic activity of daily living, *IADL* instrumental activity of daily living, *SD* standard deviation

**Variable**	**Control (no confirmed diagnosis), *n* = 12,604**	**Exposure (having confirmed diagnosis), *n* = 1261**	*P*
**Age (in years)**	76.47 (9.42)	76.59 (9.55)	0.673
**Sex (= female)**	7840 (62.2%)	784 (62.2%)	1.000
**Race/ethnicity**			
Hispanic	1020 (8.1%)	102 (8.1%)	0.999
Non-Hispanic Black	1654 (13.1%)	166 (13.2%)	
Non-Hispanic White	9930 (78.8%)	993 (78.7%)	
** Living alone (= yes)**	5855 (46.5%)	636 (50.4%)	**0.008**
** Proxy response (= yes)**	701 (5.6%)	460 (36.5%)	** < 0.001**
** Per capita household income**	5885.64 (24,921.14)	5261.22 (20,815.44)	0.319
** Had long-term care insurance (= yes)**	1748 (14.2%)	159 (13.0%)	0.281
** Number of children**	3.27 (2.06)	3.30 (2.13)	0.657
**Physical disability**			
BADL disability (= yes)	2760 (21.9%)	655 (51.9%)	** < 0.001**
IADL disability (= yes)	2274 (18.0%)	873 (69.2%)	** < 0.001**
**Receipt of BADL/IADL social support**			
Receipt of BADL social support (= yes)	1075 (8.5%)	481 (38.2%)	** < 0.001**
Receipt of IADL social support (= yes)	1828 (14.5%)	814 (64.7%)	** < 0.001**
** Score of telephone interview for cognitive status**	12.47 (5.14)	6.19 (6.18)	** < 0.001**
** Score of proxy-assessment on cognitive impairment**	2.45 (1.19)	4.15 (4.12)	** < 0.001**

### Overall results on unmet social support

**Fig. 2:**
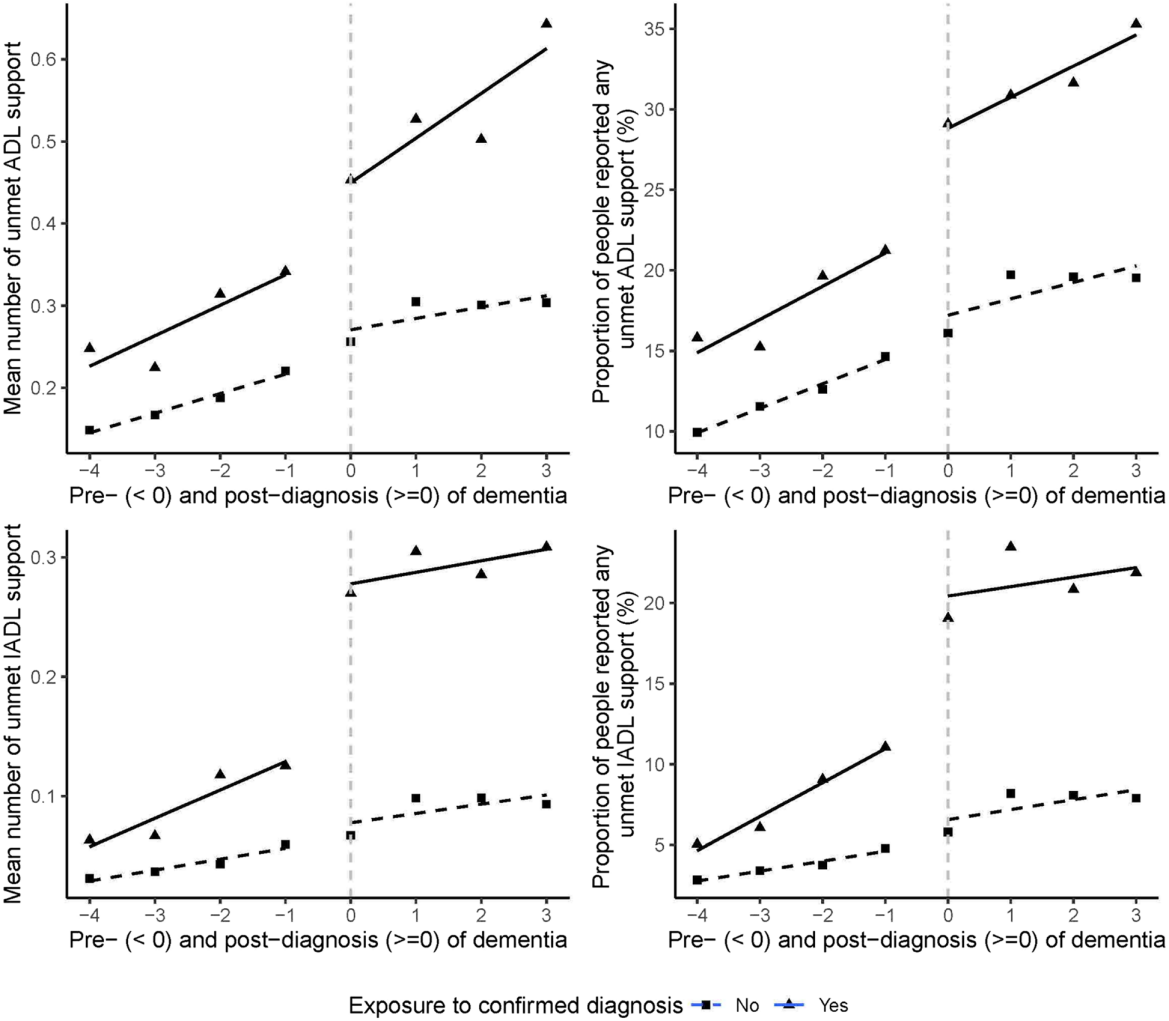
Controlled interrupted time series analysis of unmet social support in dementia diagnosis cohort (square points) and non-diagnosis cohort (triangle points), by overall. The dashed or solid lines are the model fit for two cohorts

After being diagnosed with dementia, adults in the U.S. experienced a significant step increase in the number of any unmet IADL support (Fig. 2 panel 3;Table 2, β6 = 0.10, 95% CI [0.07, 0.13]), significantly driven by the step increase in unmet needs for making phone calls (Table 2, β6 = 0.74, 95% CI [0.16, 1.33]). The step increase in the number of any unmet IADL support was followed by a significant increasing trend (Fig. 2 panel 3;Table 2, β7 = 0.02, 95% CI [0.00, 0.03]).

**Table 2:** Step effect and trend effect of dementia diagnosis on the unmet social support, by overall. Step change (β6) represents the immediate change at the diagnosis point compared to the control group, while trend change (β7) represents the change in trajectory following diagnosis versus the pre-diagnosis trend. For example, the number of unmet IADL support (β6 = 0.10) indicates an average increase of 0.10 additional unmet needs out of 5 IADL domains immediately after diagnosis, equivalent to 1 extra unmet need per 10 people diagnosed; while “on dressing” (β6 =  − 0.15) shows a log odds decrease of 0.15, representing 14% lower odds [exp(− 0.15) = 0.86] of unmet dressing support after diagnosis. Coefficients for continuous outcomes (†) represent absolute changes while binary outcomes (‡) represent log odds

Outcomes	Step change	Trend change
**Number of any unmet BADL support †**	0.00 (− 0.06, 0.06)	− 0.02 (− 0.05, 0.01)
**Having any unmet BADL support ‡**	0.10 (− 0.13, 0.33)	− 0.08 (− 0.20, 0.03)
On dressing ‡	− 0.15 (− 0.52, 0.23)	− 0.13 (− 0.33, 0.06)
On walking across a room ‡	− 0.04 (− 0.44, 0.37)	0.03 (− 0.17, 0.22)
On bathing ‡	− 0.08 (− 0.52, 0.37)	− 0.16 (− 0.39, 0.06)
On eating ‡	0.06 (− 0.50, 0.63)	− 0.22 (− 0.51, 0.06)
On getting in and out of bed ‡	− 0.39 (− 0.79, 0.01)	− 0.16 (− 0.37, 0.04)
On toileting ‡	0.23 (− 0.15, 0.62)	0.05 (− 0.13, 0.23)
**Number of any unmet IADL support †**	0.10 (0.07, 0.13) ***	0.02 (0.00, 0.03) *
**Having any unmet IADL support ‡**	0.22 (− 0.09, 0.53)	− 0.05 (− 0.20, 0.10)
On preparing a hot meal ‡	0.27 (− 0.32, 0.86)	0.00 (− 0.28, 0.29)
On shopping for groceries ‡	0.66 (− 0.04, 1.35)	0.05 (− 0.26, 0.37)
On making phone calls ‡	0.74 (0.16, 1.33) *	0.10 (− 0.17, 0.37)
On taking medications ‡	− 0.03 (− 0.72, 0.65)	− 0.36 (− 0.74, 0.02)
On managing money ‡	− 0.28 (− 0.88, 0.31)	− 0.14 (− 0.44, 0.16)

^†^Data was fitted by multi-level linear regression model, coefficients represent absolute changes in the outcome with their 95% confidence intervals. ‡Data was fitted by multi-level logistic regression, coefficients represent log odds of the outcome with their 95% confidence intervals. ***p < 0.001; **p < 0.01; *p < 0.05

### Racial/ethnic differences in unmet social support

**Fig. 3:**
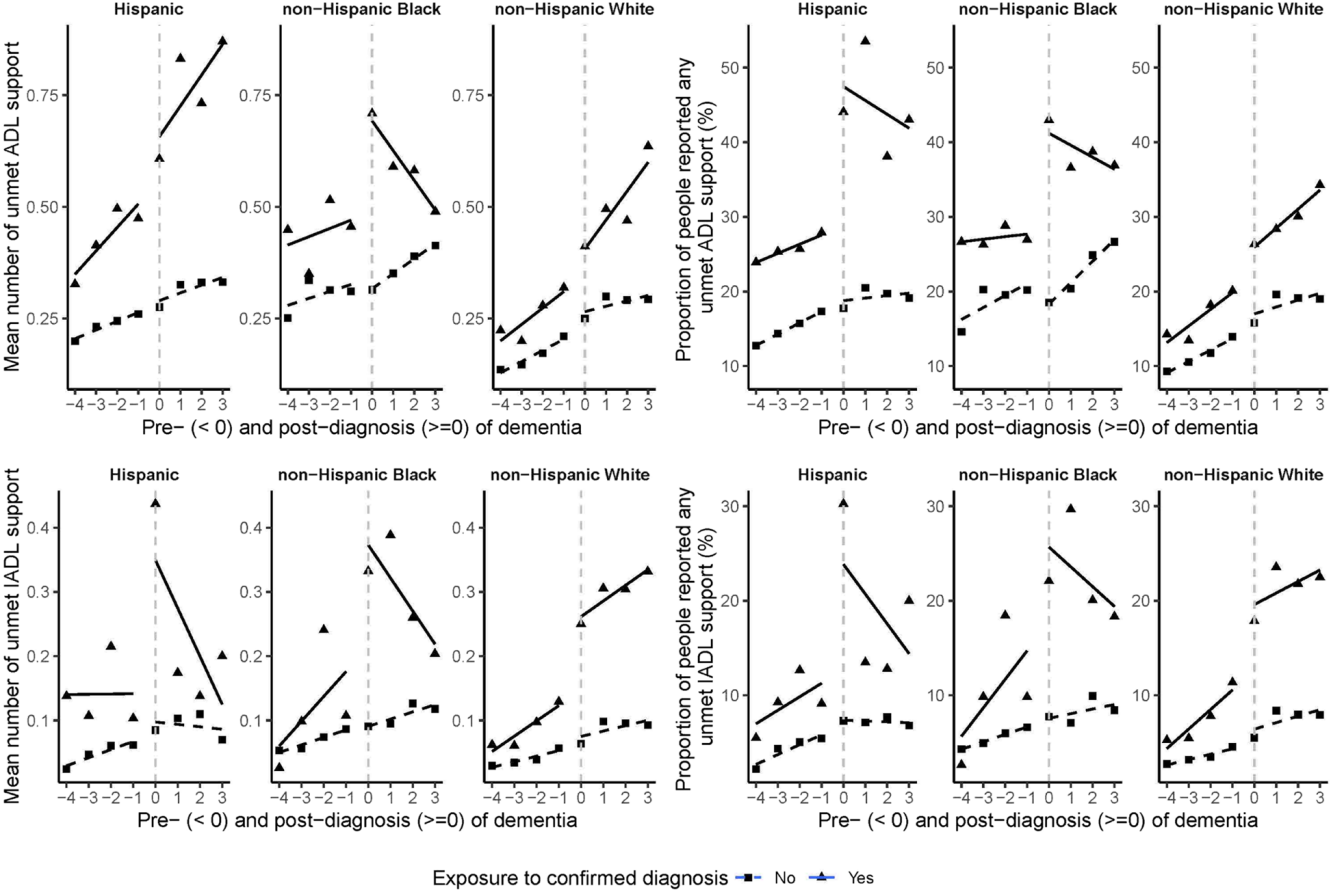
Controlled interrupted time series analysis of unmet social support in dementia diagnosis cohort (square points) and non-diagnosis cohort (triangle points), by race/ethnicity. The dashed or solid lines are the model fit for two cohorts

After being diagnosed with dementia, Hispanics experienced a significant step increase in the proportion of unmet needs for BADL support (Fig. 3 panel 2;Table 3, β6 = 0.74, 95% CI [0.03, 1.46]), significantly driven by the step increase in unmet needs for eating assistance (Table 3, β6 = 1.58, 95% CI [0.17, 2.99]). The step increases in having any unmet BADL support were followed by non-significant decreasing trends (Fig. 3 panel 2;Table 3, β7 =  − 0.05, 95% CI [− 0.40, 0.29]), while the step increase in unmet needs for eating assistance was followed by a non-significant increasing trend (Table 3, β7 = 0.03, 95% CI [− 0.59, 0.65]). Hispanics also experienced a significant step increase in the number of unmet IADL support (Fig. 3 panel 3;Table 3, β6 = 0.12, 95% CI [0.02, 0.21]), and this step increase was followed by a non-significant decrease trend (Fig. 3 panel 3;Table 3, β7 =  − 0.01, 95% CI [− 0.06, 0.03]).

**Table 3:** Step effect and trend effect of dementia diagnosis on the unmet social support, by race/ethnicity. Step change (β6) represents the immediate change at diagnosis point compared to the control group, while trend change (β7) represents the change in trajectory following diagnosis versus the pre-diagnosis trend, stratified by race/ethnicity. For example, among Hispanics, the number of unmet IADL support (β6 = 0.12) indicates an average increase of 0.12 additional unmet needs out of 5 IADL domains immediately after diagnosis, equivalent to approximately 1.2 extra unmet needs per 10 people diagnosed; while “on eating” (β6 = 1.58) shows a log odds increase of 1.58, representing nearly fivefold higher odds [exp(1.58) = 4.85] of unmet eating support after diagnosis among Hispanics. Coefficients for continuous outcomes (†) represent absolute changes while binary outcomes (‡) represent log odds

Outcomes	Hispanic		Non-Hispanic Black		Non-Hispanic White	
	Step change	Trend change	Step change	Trend change	Step change	Trend change
**Number of any unmet BADL support †**	0.10 (− 0.13, 0.33)	− 0.00 (− 0.11, 0.11)	0.15 (− 0.04, 0.34)	− 0.06 (− 0.15, 0.03)	− 0.04 (− 0.10, 0.03)	− 0.02 (− 0.05, 0.01)
**Having any unmet BADL support ‡**	0.74 (0.03, 1.46) *	− 0.05 (− 0.40, 0.29)	0.50 (− 0.07, 1.06)	− 0.07 (− 0.34, 0.20)	− 0.04 (− 0.30, 0.22)	− 0.10 (− 0.23, 0.03)
On dressing**‡**	− 0.03 (− 1.08, 1.01)	0.06 (− 0.45, 0.57)	0.26 (− 0.65, 1.17)	− 0.08 (− 0.55, 0.40)	− 0.32 (− 0.75, 0.12)	− 0.19 (− 0.43, 0.04)
On walking across a room**‡**	0.69 (− 0.67, 2.05)	− 0.10 (− 0.75, 0.54)	0.32 (− 0.62, 1.25)	− 0.34 (− 0.79, 0.12)	− 0.27 (− 0.72, 0.18)	0.06 (− 0.17, 0.28)
On bathing**‡**	0.35 (− 0.96, 1.65)	0.26 (− 0.35, 0.86)	− 0.41 (− 1.50, 0.67)	− 0.15 (− 0.71, 0.41)	0.06 (− 0.45, 0.56)	− 0.23 (− 0.50, 0.03)
On eating**‡**	1.58 (0.17, 2.99) *	0.03 (− 0.59, 0.65)	− 0.34 (− 2.02, 1.33)	− 0.68 (− 1.59, 0.24)	− 0.25 (− 0.90, 0.39)	− 0.28 (− 0.63, 0.06)
On getting in and out of bed**‡**	− 0.62 (− 1.57, 0.34)	− 0.35 (− 0.83, 0.14)	− 0.50 (− 1.43, 0.43)	− 0.01 (− 0.48, 0.46)	− 0.27 (− 0.74, 0.21)	− 0.12 (− 0.36, 0.12)
On toileting**‡**	0.61 (− 0.78, 2.00)	0.18 (− 0.44, 0.80)	1.52 (0.57, 2.47) **	− 0.00 (− 0.43, 0.42)	− 0.15 (− 0.57, 0.27)	0.08 (− 0.13, 0.28)
**Number of any unmet IADL support †**	0.12 (0.02, 0.21) *	− 0.01 (− 0.06, 0.03)	0.09 (0.00, 0.17) *	0.00 (− 0.04, 0.04)	0.11 (0.08, 0.14) ***	0.02 (0.01, 0.04) **
**Having any unmet IADL support‡**	0.62 (− 0.38, 1.63)	− 0.05 (− 0.53, 0.43)	0.14 (− 0.62, 0.90)	0.12 (− 0.24, 0.48)	0.27 (− 0.08, 0.61)	− 0.08 (− 0.25, 0.09)
On preparing a hot meal**‡**	− 0.18 (− 2.23, 1.87)	− 0.24 (− 1.35, 0.87)	− 0.62 (− 2.05, 0.81)	− 0.32 (− 1.05, 0.42)	0.28 (− 0.36, 0.92)	− 0.09 (− 0.40, 0.22)
On shopping for groceries**‡**	1.56 (− 0.63, 3.75)	0.41 (− 0.58, 1.39)	1.00 (− 0.88, 2.88)	0.51 (− 0.29, 1.31)	0.52 (− 0.25, 1.29)	0.04 (− 0.31, 0.39)
On making phone calls**‡**	− 0.38 (− 2.21, 1.46)	0.59 (− 0.20, 1.39)	1.16 (− 0.45, 2.77)	0.07 (− 0.68, 0.82)	0.83 (0.19, 1.47) *	0.04 (− 0.27, 0.35)
On taking medications**‡**	1.49 (− 0.44, 3.42)	− 0.43 (− 1.38, 0.52)	0.11 (− 1.26, 1.49)	− 1.07 (− 2.04, − 0.10) *	− 0.58 (− 1.42, 0.25)	− 0.13 (− 0.58, 0.33)
On managing money**‡**	− 0.03 (− 1.79, 1.72)	− 0.21 (− 1.10, 0.67)	0.42 (− 0.99, 1.82)	− 0.03 (− 0.69, 0.64)	− 0.51 (− 1.17, 0.15)	− 0.35 (− 0.69, − 0.01) *

^†^Data was fitted by a multi-level linear regression model; coefficients represent absolute changes in the outcome with their 95% confidence intervals. ‡Data was fitted by a multi-level logistic regression; coefficients represent log odds of the outcome with their 95% confidence intervals. ***p < 0.001; **p < 0.01; *p < 0.05

After being diagnosed with dementia, Black people experienced a significant step increase in the proportion of unmet BADL needs for toileting support (Table 3, β6 = 1.52, 95% CI [0.57, 2.47]), which was followed by a non-significant decreasing trend (Table 3, β7 <  − 0.01, 95% CI [− 0.43, 0.42]). Black people also experienced a significant step increase in the number of unmet IADL support (Fig. 3 panel 3;Table 3, β6 = 0.09, 95% CI [0.00, 0.17]), which was also followed by a non-significant increasing trend (Fig. 3 panel 3;Table 3, β7 < 0.01, 95% CI [− 0.04, 0.04]). Black people experienced a non-significant step increase in the proportion of unmet IADL needs for taking medicines support (Fig. 3 panel 4;Table 3, β6 = 0.11, 95% CI [− 1.26, 1.49]), which was followed by a significant decreasing trend (Fig. 3 panel 4;Table 3, β7 =  − 1.07, 95% CI [− 2.04, − 0.10]).

After being diagnosed with dementia, Whites experienced a significant step increase in the number of unmet IADL support (Fig. 3 panel 3;Table 3, β6 = 0.11, 95% CI [0.08, 0.14]), significantly driven by the step increase in unmet needs for making phone calls (Table 3, β6 = 0.83, 95% CI [0.19, 1.47]). The step increases in the number of unmet IADL support were followed by significant increasing trends (Fig. 3 panel 3;Table 3, β7 = 0.02, 95% CI [0.01, 0.04]), while the step increase in unmet needs for making phone calls was followed by a non-significant increasing trend (Table 3, β7 = 0.04, 95% CI [− 0.27, 0.35]). Whites experienced a non-significant step decrease in the proportion of unmet IADL needs for managing money support (Table 3, β6 =  − 0.51, 95% CI [− 1.17, 0.15]), which was followed by a significant decreasing trend (Table 3, β7 =  − 0.35, 95% CI [− 0.69, − 0.01]).

### Gender differences in unmet social support

**Fig. 4:**
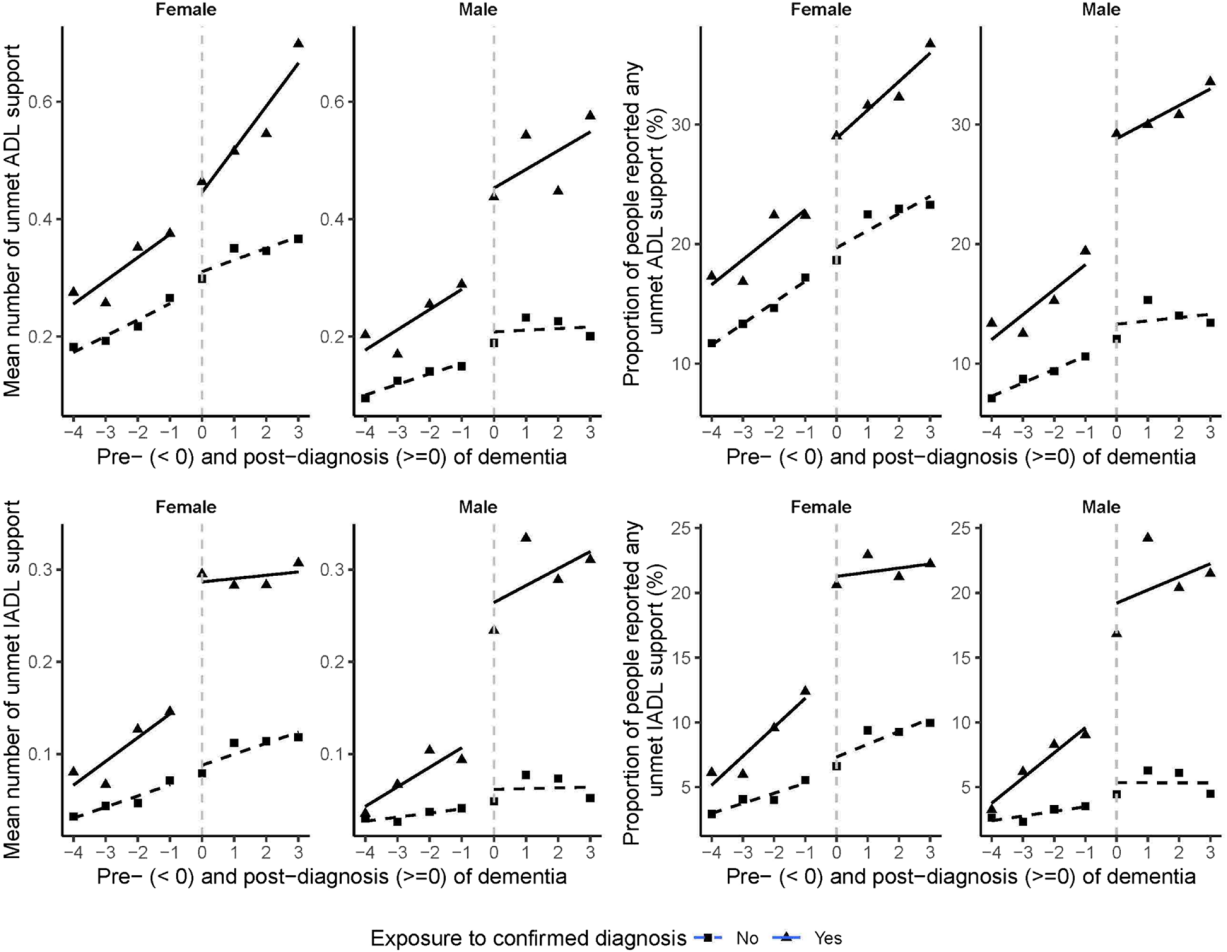
Controlled interrupted time series analysis of unmet social support in dementia diagnosis cohort (square points) and non-diagnosis cohort (triangle points), by sex. The dashed or solid lines are the model fit for two cohorts

After being diagnosed with dementia, females experienced a significant step decrease in the proportion of unmet BADL need for getting in and out of bed support (Table 4, β6 =  − 0.55, 95% CI [− 1.03, − 0.06]), which was followed by a non-significant decreasing trend over time (Table 4, β7 =  − 0.15, 95% CI [− 0.39, 0.10]). Females also experienced a significant step increase in the number of unmet IADL support (Fig. 4 panel 3; Table 4, β6 = 0.08, 95% CI [0.04, 0.11]), significantly driven by the step increase in the proportion of unmet needs for making phone calls support (Table 4, β6 = 0.96, 95% CI [0.21, 1.71]). Both the step increases in the number of unmet IADL support and in unmet support for making phone calls were followed by non-significant increasing trends over time (Table 4, β7 = 0.01, 95% CI [− 0.01, 0.03]; β7 = 0.18, 95% CI [− 0.17, 0.52], respectively), although there was a non-significant step decrease in the proportion of females with unmet IADL needs for managing money support (Table 4, β6 =  − 0.35, 95% CI [− 1.05, 0.34]), which was followed by a significant decreasing trend over time (Table 4, β7 =  − 0.45, 95% CI [− 0.82, − 0.09]).

**Table 4:** Step effect and trend effect of dementia diagnosis on the unmet social support, by sex. Step change (β6) represents the immediate change at diagnosis point compared to the control group, while trend change (β7) represents the change in trajectory following diagnosis versus the pre-diagnosis trend, stratified by sex. For example, among females, the number of unmet IADL support (β6 = 0.08) indicates an average increase of 0.08 additional unmet needs out of 5 IADL domains immediately after diagnosis, equivalent to approximately 0.8 extra unmet need per 10 women diagnosed; while among males, “on toileting” (β6 = 0.78) shows a log odds increase of 0.78, representing more than twofold higher odds [exp(0.78) = 2.18] of unmet toileting support after diagnosis. Coefficients for continuous outcomes (†) represent absolute changes while binary outcomes (‡) represent log odds

Outcomes	Female		Male	
	Step change	Trend change	Step change	Trend change
**Number of any unmet BADL support†**	− 0.02 (− 0.10, 0.06)	− 0.00 (− 0.04, 0.03)	0.06 (− 0.02, 0.14)	− 0.05 (− 0.09, − 0.01) *
**Having any unmet BADL support‡**	0.09 (− 0.19, 0.37)	− 0.01 (− 0.15, 0.12)	0.12 (− 0.25, 0.49)	− 0.20 (− 0.38, − 0.01) *
On dressing**‡**	0.08 (− 0.42, 0.57)	− 0.08 (− 0.34, 0.18)	− 0.45 (− 0.99, 0.10)	− 0.18 (− 0.47, 0.11)
On walking across a room**‡**	− 0.18 (− 0.67, 0.31)	0.09 (− 0.15, 0.32)	− 0.05 (− 0.70, 0.60)	− 0.27 (− 0.59, 0.05)
On bathing**‡**	− 0.02 (− 0.54, 0.50)	− 0.17 (− 0.43, 0.10)	0.02 (− 0.77, 0.80)	− 0.12 (− 0.52, 0.28)
On eating**‡**	0.16 (− 0.53, 0.86)	− 0.16 (− 0.50, 0.18)	− 0.18 (− 1.09, 0.72)	− 0.25 (− 0.74, 0.24)
On getting in and out of bed**‡**	− 0.55 (− 1.03, − 0.06) *	− 0.15 (− 0.39, 0.10)	0.07 (− 0.57, 0.72)	− 0.13 (− 0.45, 0.19)
On toileting**‡**	− 0.05 (− 0.48, 0.38)	0.09 (− 0.11, 0.30)	0.78 (0.03, 1.53) *	0.03 (− 0.31, 0.37)
**Number of any unmet IADL support†**	0.08 (0.04, 0.11) ***	0.01 (− 0.01, 0.03)	0.14 (0.10, 0.18) ***	0.02 (0.00, 0.04) *
**Having any unmet IADL support‡**	0.15 (− 0.22, 0.51)	− 0.05 (− 0.23, 0.13)	0.43 (− 0.08, 0.94)	− 0.07 (− 0.32, 0.18)
On preparing a hot meal**‡**	− 0.25 (− 0.90, 0.39)	− 0.10 (− 0.43, 0.22)	0.90 (− 0.33, 2.14)	− 0.37 (− 1.00, 0.25)
On shopping for groceries**‡**	0.43 (− 0.41, 1.27)	0.06 (− 0.34, 0.45)	0.91 (− 0.24, 2.07)	0.23 (− 0.30, 0.75)
On making phone calls**‡**	0.96 (0.21, 1.71) *	0.18 (− 0.17, 0.52)	0.63 (− 0.22, 1.47)	0.04 (− 0.35, 0.44)
On taking medications**‡**	− 0.25 (− 1.05, 0.54)	− 0.32 (− 0.77, 0.13)	0.05 (− 1.13, 1.24)	− 0.48 (− 1.13, 0.18)
On managing money**‡**	− 0.35 (− 1.05, 0.34)	− 0.45 (− 0.82, − 0.09) *	− 0.07 (− 1.04, 0.90)	0.02 (− 0.44, 0.49)

^†^Data was fitted by multi-level linear regression model, coefficients represent absolute changes in the outcome with their 95% confidence intervals. ‡Data was fitted by multi-level logistic regression, coefficients represent log odds of the outcome with their 95% confidence intervals. ***p < 0.001; **p < 0.01; *p < 0.05

After being diagnosed with dementia, males experienced non-significant step increases in the number of unmet BADL support (Fig. 4 panel 1; Table 4, β6 = 0.06, 95% CI [− 0.02, 0.14]) and in the proportion of unmet BADL support (Fig. 4 panel 2; Table 4, β6 = 0.12, 95% CI [− 0.25, 0.49]), yet both were followed by a significant decreasing trend over time (Fig. 4 panels 1 and 2; Table 4, β7 =  − 0.05, 95% CI [− 0.09, − 0.01]; β7 =  − 0.20, 95% CI [− 0.38, − 0.01], respectively). Males experienced a significant step increase in the proportion of unmet BADL support for toileting (Table 4, β6 = 0.78, 95% CI [0.03, 1.53]), which was followed by a non-significant increasing trend over time (Table 4, β7 = 0.03, 95% CI [− 0.31, 0.37]). Males also experienced a significant step increase in the number of unmet IADL support (Fig. 4 panel 3; Table 4, β6 = 0.14, 95% CI [0.10, 0.18]), which was followed by a significant increasing trend (Fig. 4 panel 3; Table 4, β7 = 0.02, 95% CI [0.00, 0.04]).

### Potential reasons on unmet social support

The supplementary analysis (Additional file 1: Table S1–S3) on physical disability and social support indicated that the above step increases in unmet social support was primarily due to the step increase in the disability greater than the step increase in the corresponding social support. However, there were two exceptions: among black people, the step increase in unmet needs for toileting support resulted from the step increase in disability in toileting (β6 = 0.91, 95% CI [0.15, 1.66]) and step decrease in social support of toileting (β6 =  − 1.09, 95% CI [− 2.30, 0.13]) (Additional file 1: Table S2); among females the step decrease in unmet needs for getting in and out of bed resulted from the step decrease in disability in getting in and out of bed (β6 =  − 0.28, 95% CI [− 0.63, 0.07]) greater than the step decrease in social support of getting in and out of bed (β6 =  − 0.23, 95% CI [− 0.75, 0.28]) (Additional file 1: Table S3).

### Sensitivity analysis

The sensitivity analysis by matching the control cohort based on all general people (Additonal file 1: Figure S1–S3 and Table S4–S6) confirms the consistency of the above main results.

## Discussion

### Statement of principal findings

This large, nationally representative matched ambidirectional cohort study provided novel insights into the discontinuity of unmet social support needs before and after a dementia diagnosis. With a rigorous study design to detect causal effects in time series data, the findings indicated that dementia diagnosis was associated with rises in unmet social support needs, with variations by race/ethnicity and gender. The increased unmet needs primarily resulted from a greater increase in physical disabilities than an increase in corresponding social supports after diagnosis. After the dementia diagnosis, Hispanics experienced significant increases in unmet BADL and IADL needs, particularly for eating assistance; Black people experienced more unmet needs for toileting support and IADL support; and Whites experienced a significant rise in unmet IADL support, especially for making phone calls. Gender-wise, females showed a significant decrease in unmet BADL support for getting in and out of bed, but an increase in unmet IADL support, particularly making phone calls; and Males experienced increases in unmet toileting support and IADL support. Over time, the increase in unmet social support needs triggered by dementia diagnosis does not significantly return to its original levels, but had significant or insignificant increase trends.

### Interpretation

The universal rise in unmet IADL assistance across sociodemographic subgroups after a dementia diagnosis suggests that the point of diagnosis might not be associated with optimal care planning. While our diagnosed group showed greater cognitive impairment than the control group, our study design focusing on trends before and after diagnosis allows us to isolate the effect of the diagnosis itself, independent of absolute cognitive status. This finding represents a key contribution to the literature, revealing that rather than serving as an effective intervention point to align support with needs, diagnosis is associated with a widening support gap. The further exploration of this rising revealed a mismatch between sharply rising disability levels and inadequately compensatory social support after a dementia diagnosis. Therefore, this post-diagnosis surge in unmet IADL assistance likely stems from fragmented coordination between formal and informal networks failing to meet the escalating needs of people with dementia as they navigate life in their communities following the diagnosis. This aligns with prior evidence that dementia is associated with marked increased disability [1, 42], but runs counter to our desire to leverage diagnosis as a platform to activate interdisciplinary health services alongside proactive family/friend capacity-building from an early stage [5, 10]. Specifically, patients across subgroups face surging unmet IADL needs immediately post-diagnosis, reflecting system-wide inability to put in place sustainable assistance. Communication deficiencies between specialist care providers [14], exclusion of patients and caregivers from care planning [43, 44], and instability of informal care commitments over time [45] could collectively leave patients stranded and unable to bridge the post-diagnostic disability-need gap. Additionally, individual-level barriers—including inadequate economic resources, complex insurance procedures, insufficient insurance coverage, lack of anticipatory guidance for caregivers in pre-diagnosis, and delayed access to team-based supports [3, 46, 47]—could further compound these post-diagnosis discontinuities. Furthermore, patient and family education, as well as awareness of available services, may rely solely on the initiative of family caregivers or the newly diagnosed individual living with dementia. This could lead to delays in care planning. Therefore, a key research outcome could involve examining the optimal timeframe for delivering education and initiating care planning following a dementia diagnosis. Our findings underscore the necessity to integrate formal health services and informal care into a long-term, patient-centered care ecosystem and to use diagnosis as a window to continually assess and proactively bridge evolving social support needs. Professionals should increase referrals for social support after diagnosis, primarily from a primary care provider or specialist visit. Additionally, our findings highlight the need to continually monitor the needs of people with dementia and repeatedly assess caregiver resilience and formal care barriers to activate better integrated and adeptly responsive assistance as disability profiles shift over time [11].

There are concerning racial/ethnic inequities in unmet support needs triggered by a dementia diagnosis. The disproportionate rise in social support gaps for essential daily activities among Hispanics, Black people, and Whites likely stems from subgroup variations in a number of variables including access barriers and economic constraints [3, 46, 47]. For instance, the sharp escalations in assistance gaps for essential BADLs among Hispanic and Black groups indicate more complex progression perhaps due to delayed diagnosis [46, 48]. Supported by prior evidence on hierarchical loss patterns, the specific activities—eating and toileting—typically manifest in later-stage disease [49, 50]. This mirrors past research showing racial/ethnic minorities facing barriers to timely diagnosis and specialized services access [46, 48]. Furthermore, we hypothesized that the trend of unmet social support would decrease or not significantly change; however, Whites showed continuously rising unmet IADL support over time, contrasting with plateauing gaps for Black people and Hispanics—signalling inequities in care network responses. In the US, informal care from families and friends is commonly the primary source of support for older adults with cognitive impairment, and ethnic minorities devote more time to informal care than those of White people [9]. The gradually increasing IADL support gaps within Whites may stem from the strained capacity of earlier-diagnosed informal networks to continually expand assistance along unpredictable trajectories. Our findings in racial/ethnic disparities emphasized the importance to implement culturally appropriate strategies for care-planning, and enhance equitable diagnosis and care access for disadvantaged groups in US populations.

We also found some gender disparities in unmet support needs triggered by a dementia diagnosis. Females showed a significant decrease in unmet BADL support for getting in and out of bed, but no significant change in unmet BADL support over time. This aligns with evidence on females having wider social connections though losing accessible formal supports as dementia advances [51–53]. For males, despite the immediate increase in unmet IADL support, they also had a significant increasing trend in unmet IADL support. In contrast to females, males displayed significant decreasing trends in unmet BADL support over time, signalling possible norms of self-reliance being outweighed by disability despite some stigma persisting around personal care assistance [54, 55]. However, rising gaps in complex IADL tasks among males highlight eventual accelerating strains on solitary coping capacity. These findings enrich existing knowledge on gender variations in support mobilization and caregiver burden. While females may have more success initially eliciting assisted daily living support, males and females both confront demands exceeding the capability of informal networks as the condition progresses. Targeted interventions promoting equitable access to formal services alongside sensitization around masculinity/femininity in care receipt may help address these gendered trajectory disparities after a dementia diagnosis.

Despite some significant decreasing trends in unmet support over time, the increase in unmet social support needs triggered by dementia diagnosis does not significantly return to their original levels before diagnosis. The inability to mitigate initially triggered support gaps spotlights systemic inadequacies in matching ongoing neurodegenerative decline. Reasons likely include unsustainable reliance on informal caregivers confronting exponential stressors, gaps in anticipatory education on disease progression, and narrow eligibility criteria for formal services that fail to accommodate incremental disabling [1, 3, 42, 45–47, 56, 57]. The expanding trajectory of unmet basic and instrumental support needs found in this study predicts potentially intensifying adverse outcomes [17, 18, 22, 27]. This underscores the need to implement layered supports spanning familial, provider, and policy levels—to continually track and adapt care as dementia-related disabilities progressively and irreversibly worsen, even many years post-diagnosis. The point of diagnosis provides an opportunity to assess and address care needs, with a focus on addressing all current care needs and putting plans in place to continue to monitor difficulties and mitigate them with increased support.

## Strengths and limitations

To the best of our knowledge, this is the first study worldwide to explore the continuity of unmet social support before and after the diagnosis of dementia. This study employed an ambidirectional cohort design, and our two cohorts were matched based on key demographics and limited to those with sufficient pre- and post-diagnosis data. This approach strengthened the homogeneity of our two cohorts and enhanced internal validity. The data was analyzed with the CITS model, which enabled the causal inference on unmet social support resulting from the dementia diagnosis.

Some limitations should be considered when interpreting the results. First, the study’s focus on a U.S. population limits the generalizability of its findings to other cultural and healthcare contexts. The reliance on self-reported and proxy-reported data introduces potential biases, particularly in the assessment of cognitive impairment and social support needs. Although the interrupted time series method is a causal inference model, it is still limited by the non-randomized design. Second, the HRS does not provide the exact date of dementia diagnosis, but rather the date of the next wave of data collection following the diagnosis. As a result, the actual diagnosis could have occurred earlier than the identified date point, potentially leading to an underestimation of the step effects. Third, there is a significant difference in cognitive performance between the diagnosed and control groups at baseline, with the diagnosed group showing lower scores on standardized cognitive assessments. While our interrupted time series design focuses on analyzing trends rather than absolute differences between groups, this baseline cognitive disparity may influence the interpretation of our findings. The greater cognitive impairment in the diagnosed group could potentially contribute to different patterns of support needs independent of the diagnosis event itself. However, by using each group as its own control in the pre-post analysis, our methodology partially mitigates this concern by focusing on changes relative to each group’s own baseline. Fourth, the HRS dataset does not include information on urban versus rural place of residence, which limits our ability to examine how geographic location and associated differences in community support infrastructure may influence patterns of unmet social support needs following dementia diagnosis. Finally, while our study examined patterns of unmet support across racial/ethnic and gender subgroups, we did not formally test for statistically significant differences between these groups. This approach was taken considering several data limitations: the sample sizes for certain racial/ethnic subgroups (particularly Hispanics and Blacks) were relatively small which could limit statistical power for complex interaction testing; and our multilevel CITS models were already complex with numerous parameters. Future research with larger, more frequent data collection should employ formal interaction testing to quantitatively assess disparities in care continuity across demographic groups, which would further strengthen evidence for targeted interventions. The current stratified analyses, however, still provide valuable insights into the distinct patterns of care discontinuity experienced by different populations following a dementia diagnosis.

## Conclusions

This nationally representative, longitudinal investigation reveals a concerning system-wide vulnerability in ensuring continuity of social support following a dementia diagnosis. Moving beyond merely documenting increased care needs in dementia, our study uniquely identifies the diagnosis event itself as a critical but currently ineffective transition point in the care continuum. Leveraging a rigorous quasi-experimental design, the study underscores post-diagnosis surges in disability assistance gaps across sociodemographic groups—countering assumptions of care stabilization resulting from diagnosis and assessment. The findings describe increasing gaps in care as needs accelerate. Additionally, subgroup disparities indicate inequities in meeting care needs in different sexes and racial groups. Overall, these outcomes highlight imperatives across policy, health practice, and research to implement structural reforms—fostering layered formal-informal scaffolds, proactive alignment of capacities to advancing disabilities, and transitional protocols bridging care settings—to promote more equitable, effective, and continuous assistance for people living with dementia.

## Supplementary Information

Additional file 1: Table S1: Step effect and trend effect of dementia diagnosis on the physical disability and corresponding social support, by overall

Additional file 2: Table S2: Step effect and trend effect of dementia diagnosis on the physical disability and corresponding social support, by race/ethnicity

Additional file 3: Table S3: Step effect and trend effect of dementia diagnosis on the physical disability and corresponding social support, by sex

Additional file 4: Table S4: Step effect and trend effect of dementia diagnosis on the unmet social support, by overall, matching the control cohort based on all general people

Additional file 5: Table S5: Step effect and trend effect of dementia diagnosis on the unmet social support, by race/ethnicity, matching the control cohort based on all general people

Additional file 6: Table S6: Step effect and trend effect of dementia diagnosis on the unmet social support, by sex, matching the control cohort based on all general people

Additional file 7: Figure S1: Controlled interrupted time series analysis of unmet social support in dementia diagnosis cohortand non-diagnosis cohort, by overall, matching the control cohort based on all general people

Additional file 8: Figure S2: Controlled interrupted time series analysis of unmet social support in dementia diagnosis cohortand non-diagnosis cohort, by race/ethnicity, matching the control cohort based on all general people

Additional file 9: Figure S3: Controlled interrupted time series analysis of unmet social support in dementia diagnosis cohortand non-diagnosis cohort, by sex, matching the control cohort based on all general people

## Data Availability

The data are publicly available and can be accessed here (https://hrsdata.isr.umich.edu/data-products/rand).

## Abbreviations

HRS: Health and Retirement Survey
TICS: Telephone Interview for Cognitive Status
IADL: Instrumental activities of daily living
BADL: Basic activities of daily living
CITS: Controlled interrupted time series
CIs: Confidence intervals
