# Supplementary material for: Discontinuity of social support among US adults with cognitive impairment before and after the confirmed diagnosis of dementia: a matched ambidirectional cohort study
Source: BMC Med. 2025 Jul 15;23:428. doi: 10.1186/s12916-025-04264-y (PMC12265323; doi:10.1186/s12916-025-04264-y)
Supplement: Supplementary file 1 — Additional file 1: Table S1: Step effect and trend effect of dementia diagnosis on the physical disability and corresponding social support, by overall [file 12916_2025_4264_MOESM1_ESM.docx]

**Table S1. Step effect and trend effect of dementia diagnosis on the physical disability and corresponding social support, by overall**.

| **Outcomes** | **Step change** | **Trend change** |
| --- | --- | --- |
| **Physical disability** | | |
| **Number of any BADL disabilities †** | 0.66 (0.56, 0.76) *** | 0.15 (0.10, 0.20) *** |
| **Having any BADL disabilities‡** | 0.45 (0.24, 0.66) *** | -0.00 (-0.11, 0.11) |
| On dressing‡ | 0.38 (0.12, 0.64) ** | 0.06 (-0.07, 0.18) |
| On walking across a room‡ | 0.37 (0.06, 0.68) * | 0.09 (-0.06, 0.24) |
| On bathing‡ | 0.59 (0.29, 0.89) *** | -0.01 (-0.16, 0.14) |
| On eating‡ | 0.40 (-0.01, 0.82) | 0.01 (-0.19, 0.21) |
| On getting in and out of bed‡ | 0.10 (-0.21, 0.40) | -0.01 (-0.15, 0.14) |
| On toileting‡ | 0.52 (0.21, 0.84) *** | 0.10 (-0.04, 0.25) |
| **Number of any IADL disabilities †** | 1.15 (1.07, 1.22) *** | -0.11 (-0.15, -0.07) *** |
| **Having any IADL disabilities‡** | 0.75 (0.52, 0.97) *** | -0.29 (-0.41, -0.18) *** |
| On preparing a hot meal‡ | 0.88 (0.57, 1.19) *** | -0.36 (-0.52, -0.20) *** |
| On shopping for groceries‡ | 0.84 (0.57, 1.11) *** | -0.20 (-0.33, -0.06) ** |
| On making phone calls‡ | 0.83 (0.48, 1.17) *** | -0.17 (-0.34, 0.01) |
| On taking medications‡ | 0.54 (0.17, 0.90) ** | -0.29 (-0.48, -0.11) ** |
| On managing money‡ | 0.79 (0.50, 1.08) *** | -0.48 (-0.63, -0.33) *** |
| **Social support** | | |
| **Number of receipt of any BADL support †** | 0.66 (0.58, 0.73) *** | 0.17 (0.13, 0.21) *** |
| **Receipt of any BADL support‡** | 0.41 (0.13, 0.70) ** | -0.02 (-0.17, 0.12) |
| On dressing‡ | 0.40 (0.05, 0.75) * | 0.10 (-0.07, 0.27) |
| On walking across a room‡ | 0.44 (-0.01, 0.90) | 0.12 (-0.11, 0.35) |
| On bathing‡ | 0.75 (0.35, 1.15) *** | 0.06 (-0.15, 0.26) |
| On eating‡ | 0.42 (-0.17, 1.02) | 0.15 (-0.13, 0.43) |
| On getting in and out of bed‡ | 0.42 (-0.05, 0.89) | 0.16 (-0.07, 0.39) |
| On toileting‡ | 0.47 (-0.12, 1.06) | 0.16 (-0.13, 0.46) |
| **Number of receipt of any IADL support †** | 1.04 (0.97, 1.11) *** | -0.13 (-0.16, -0.09) *** |
| **Receipt of any IADL support‡** | 0.74 (0.50, 0.97) *** | -0.31 (-0.43, -0.19) *** |
| On preparing a hot meal‡ | 0.65 (0.31, 1.00) *** | -0.49 (-0.66, -0.31) *** |
| On shopping for groceries‡ | 0.69 (0.41, 0.97) *** | -0.25 (-0.39, -0.10) *** |
| On making phone calls‡ | 0.39 (-0.00, 0.77) | -0.40 (-0.60, -0.20) *** |
| On taking medications‡ | 0.39 (-0.06, 0.85) | -0.27 (-0.51, -0.04) * |
| On managing money‡ | 0.80 (0.48, 1.12) *** | -0.53 (-0.70, -0.37) *** |

† Data was fitted by multi-level linear regression model, coefficients represent absolute changes in the outcome with their 95% confidence intervals. ‡ Data was fitted by multi-level logistic regression, coefficients represent log odds of the outcome with their 95% confidence intervals. *** p < 0.001; ** p < 0.01; * p < 0.05.
