# Supplementary material for: Discontinuity of social support among US adults with cognitive impairment before and after the confirmed diagnosis of dementia: a matched ambidirectional cohort study
Source: BMC Med. 2025 Jul 15;23:428. doi: 10.1186/s12916-025-04264-y (PMC12265323; doi:10.1186/s12916-025-04264-y)
Supplement: Supplementary file 2 — Additional file 2: Table S2: Step effect and trend effect of dementia diagnosis on the physical disability and corresponding social support, by race/ethnicity [file 12916_2025_4264_MOESM2_ESM.docx]

**Table S2. Step effect and trend effect of dementia diagnosis on the physical disability and corresponding social support, by race/ethnicity**.

| **Outcomes** | **Hispanic** | | **Non-Hispanic Black** | | **Non-Hispanic White** | |
| --- | --- | --- | --- | --- | --- | --- |
|  | Step change | Trend change | Step change | Trend change | Step change | Trend change |
| **Physical disability** | | | | | | |
| **Number of any BADL disabilities †** | 1.06 (0.70, 1.43) *** | -0.10 (-0.28, 0.08) | 0.61 (0.32, 0.90) *** | 0.10 (-0.04, 0.24) | 0.66 (0.56, 0.76) *** | 0.18 (0.13, 0.24) *** |
| **Having any BADL disabilities‡** | 1.06 (0.38, 1.75) ** | -0.08 (-0.41, 0.25) | 0.56 (0.03, 1.10) * | 0.02 (-0.25, 0.29) | 0.44 (0.20, 0.68) *** | 0.00 (-0.12, 0.13) |
| On dressing‡ | 0.91 (0.18, 1.64) * | -0.12 (-0.46, 0.22) | 0.37 (-0.22, 0.97) | 0.09 (-0.19, 0.37) | 0.38 (0.08, 0.68) * | 0.02 (-0.13, 0.17) |
| On walking across a room‡ | 1.37 (0.37, 2.37) ** | 0.06 (-0.40, 0.51) | 0.21 (-0.49, 0.92) | -0.16 (-0.49, 0.18) | 0.17 (-0.17, 0.52) | 0.07 (-0.10, 0.25) |
| On bathing‡ | 0.91 (0.01, 1.81) * | -0.17 (-0.59, 0.25) | 0.17 (-0.51, 0.86) | -0.02 (-0.35, 0.31) | 0.69 (0.35, 1.03) *** | 0.01 (-0.16, 0.18) |
| On eating‡ | 1.87 (0.75, 2.99) *** | -0.01 (-0.50, 0.48) | 0.68 (-0.39, 1.75) | -0.14 (-0.63, 0.35) | 0.15 (-0.32, 0.61) | -0.04 (-0.28, 0.20) |
| On getting in and out of bed‡ | -0.01 (-0.77, 0.76) | -0.34 (-0.71, 0.04) | -0.09 (-0.77, 0.58) | -0.00 (-0.32, 0.31) | 0.12 (-0.24, 0.48) | 0.05 (-0.12, 0.22) |
| On toileting‡ | 1.07 (0.06, 2.07) * | -0.06 (-0.52, 0.40) | 0.91 (0.15, 1.66) * | -0.00 (-0.34, 0.34) | 0.34 (-0.01, 0.70) | 0.11 (-0.06, 0.28) |
| **Number of any IADL disabilities †** | 1.23 (0.97, 1.50) *** | -0.29 (-0.42, -0.16) *** | 1.05 (0.84, 1.25) *** | -0.12 (-0.22, -0.02) * | 1.20 (1.12, 1.28) *** | -0.10 (-0.14, -0.05) *** |
| **Having any IADL disabilities‡** | 0.97 (0.27, 1.67) ** | -0.20 (-0.54, 0.14) | 1.05 (0.49, 1.62) *** | -0.23 (-0.52, 0.07) | 0.69 (0.44, 0.94) *** | -0.36 (-0.49, -0.23) *** |
| On preparing a hot meal‡ | 0.74 (-0.21, 1.70) | -0.41 (-0.87, 0.06) | 0.66 (-0.03, 1.35) | -0.25 (-0.59, 0.09) | 0.95 (0.60, 1.31) *** | -0.41 (-0.60, -0.23) *** |
| On shopping for groceries‡ | 1.43 (0.62, 2.24) *** | -0.09 (-0.47, 0.29) | 0.63 (0.01, 1.24) * | 0.05 (-0.26, 0.36) | 0.87 (0.56, 1.18) *** | -0.29 (-0.45, -0.13) *** |
| On making phone calls‡ | 0.31 (-0.55, 1.16) | -0.28 (-0.69, 0.14) | 0.64 (-0.23, 1.51) | -0.41 (-0.85, 0.03) . | 0.98 (0.59, 1.37) *** | -0.15 (-0.34, 0.05) |
| On taking medications‡ | 0.45 (-0.48, 1.38) | -0.35 (-0.81, 0.10) | 0.51 (-0.36, 1.39) | -0.83 (-1.29, -0.37) *** | 0.51 (0.10, 0.92) * | -0.32 (-0.52, -0.11) ** |
| On managing money‡ | 1.27 (0.42, 2.12) ** | -0.33 (-0.74, 0.08) | 0.76 (0.07, 1.46) * | -0.53 (-0.88, -0.18) ** | 0.57 (0.24, 0.89) *** | -0.66 (-0.83, -0.48) *** |
| **Social support** | | | | | | |
| **Number of receipt of any BADL support †** | 0.96 (0.71, 1.21) *** | -0.10 (-0.22, 0.02) | 0.47 (0.27, 0.67) *** | 0.16 (0.06, 0.25) ** | 0.69 (0.62, 0.77) *** | 0.20 (0.16, 0.24) *** |
| **Receipt of any BADL support‡** | 0.78 (-0.05, 1.61) . | -0.11 (-0.50, 0.28) | 0.21 (-0.42, 0.85) | -0.11 (-0.43, 0.20) | 0.53 (0.20, 0.86) ** | -0.00 (-0.17, 0.17) |
| On dressing‡ | 1.09 (0.18, 1.99) * | -0.18 (-0.60, 0.24) | 0.22 (-0.51, 0.94) | 0.00 (-0.35, 0.35) | 0.48 (0.07, 0.90) * | 0.05 (-0.15, 0.26) |
| On walking across a room‡ | 1.62 (0.23, 3.02) * | 0.29 (-0.35, 0.92) | -0.10 (-1.07, 0.88) | -0.05 (-0.52, 0.41) | 0.16 (-0.35, 0.68) | 0.00 (-0.27, 0.27) |
| On bathing‡ | 0.76 (-0.43, 1.94) | -0.50 (-1.07, 0.06) | 0.41 (-0.41, 1.24) | 0.04 (-0.36, 0.43) | 0.72 (0.25, 1.18) ** | 0.06 (-0.18, 0.31) |
| On eating‡ | 1.57 (-0.17, 3.30) | 0.12 (-0.64, 0.88) | 0.94 (-0.48, 2.36) | 0.13 (-0.48, 0.74) | 0.27 (-0.38, 0.91) | 0.02 (-0.30, 0.35) |
| On getting in and out of bed‡ | 0.48 (-0.60, 1.56) | -0.21 (-0.74, 0.31) | -0.06 (-1.04, 0.93) | -0.06 (-0.51, 0.40) | 0.22 (-0.35, 0.80) | 0.16 (-0.13, 0.46) |
| On toileting‡ | 0.95 (-0.48, 2.38) | -0.15 (-0.81, 0.50) | -1.09 (-2.30, 0.13) | -0.51 (-1.08, 0.06) | 0.43 (-0.29, 1.15) | 0.10 (-0.27, 0.47) |
| **Number of receipt of any IADL support †** | 1.13 (0.89, 1.37) *** | -0.27 (-0.38, -0.15) *** | 0.95 (0.77, 1.13) *** | -0.12 (-0.21, -0.03) ** | 1.08 (1.01, 1.15) *** | -0.12 (-0.16, -0.08) *** |
| **Receipt of any IADL support‡** | 0.87 (0.16, 1.59) * | -0.22 (-0.57, 0.12) | 0.89 (0.32, 1.47) ** | -0.26 (-0.56, 0.03) | 0.71 (0.45, 0.98) *** | -0.39 (-0.54, -0.25) *** |
| On preparing a hot meal‡ | 0.66 (-0.40, 1.71) | -0.46 (-0.97, 0.04) | 0.77 (0.02, 1.52) * | -0.25 (-0.62, 0.11) | 0.68 (0.28, 1.08) *** | -0.57 (-0.79, -0.35) *** |
| On shopping for groceries‡ | 1.24 (0.40, 2.08) ** | -0.16 (-0.55, 0.23) | 0.51 (-0.13, 1.14) | -0.08 (-0.39, 0.24) | 0.71 (0.39, 1.04) *** | -0.36 (-0.53, -0.19) *** |
| On making phone calls‡ | 0.23 (-0.70, 1.17) | -0.54 (-1.00, -0.07) * | 0.15 (-0.84, 1.14) | -0.66 (-1.18, -0.15) * | 0.45 (0.01, 0.89) * | -0.43 (-0.66, -0.21) *** |
| On taking medications‡ | 0.10 (-0.96, 1.17) | -0.27 (-0.80, 0.26) | 0.42 (-0.70, 1.53) | -1.02 (-1.63, -0.42) *** | 0.46 (-0.04, 0.95) . | -0.35 (-0.60, -0.09) ** |
| On managing money‡ | 1.27 (0.34, 2.19) ** | -0.28 (-0.71, 0.16) | 0.55 (-0.22, 1.32) | -0.68 (-1.08, -0.29) *** | 0.58 (0.23, 0.94) *** | -0.71 (-0.90, -0.52) *** |

† Data was fitted by multi-level linear regression model, coefficients represent absolute changes in the outcome with their 95% confidence intervals. ‡ Data was fitted by multi-level logistic regression, coefficients represent log odds of the outcome with their 95% confidence intervals. *** p < 0.001; ** p < 0.01; * p < 0.05..
