# Supplementary material for: Discontinuity of social support among US adults with cognitive impairment before and after the confirmed diagnosis of dementia: a matched ambidirectional cohort study
Source: BMC Med. 2025 Jul 15;23:428. doi: 10.1186/s12916-025-04264-y (PMC12265323; doi:10.1186/s12916-025-04264-y)
Supplement: Supplementary file 3 — Additional file 3: Table S3: Step effect and trend effect of dementia diagnosis on the physical disability and corresponding social support, by sex [file 12916_2025_4264_MOESM3_ESM.docx]

**Table S3. Step effect and trend effect of dementia diagnosis on the physical disability and corresponding social support, by sex**.

| **Outcomes** | **Female** | | **Male** | |
| --- | --- | --- | --- | --- |
|  | Step change | Trend change | Step change | Trend change |
| **Physical disability** | | | | |
| **Number of any BADL disabilities †** | 0.45 (0.33, 0.58) *** | 0.20 (0.14, 0.26) *** | 0.80 (0.67, 0.93) *** | 0.04 (-0.02, 0.11) |
| **Having any BADL disabilities‡** | 0.42 (0.16, 0.68) ** | 0.08 (-0.06, 0.21) | 0.49 (0.15, 0.83) ** | -0.11 (-0.28, 0.06) |
| On dressing‡ | 0.28 (-0.04, 0.59) | 0.11 (-0.05, 0.26) | 0.53 (0.12, 0.94) * | -0.10 (-0.30, 0.11) |
| On walking across a room‡ | 0.01 (-0.35, 0.36) | 0.17 (-0.01, 0.34) . | 0.55 (0.01, 1.09) * | -0.24 (-0.51, 0.02) |
| On bathing‡ | 0.44 (0.10, 0.78) * | 0.01 (-0.16, 0.18) | 0.82 (0.25, 1.38) ** | -0.06 (-0.34, 0.23) |
| On eating‡ | 0.39 (-0.09, 0.88) | 0.06 (-0.17, 0.29) | 0.47 (-0.22, 1.16) | -0.09 (-0.44, 0.27) |
| On getting in and out of bed‡ | -0.28 (-0.63, 0.07) | -0.00 (-0.17, 0.17) | 0.64 (0.13, 1.16) * | -0.03 (-0.28, 0.21) |
| On toileting‡ | 0.17 (-0.19, 0.52) | 0.18 (0.01, 0.35) * | 1.06 (0.43, 1.69) *** | -0.11 (-0.40, 0.18) |
| **Number of any IADL disabilities †** | 1.05 (0.95, 1.15) *** | -0.14 (-0.19, -0.09) *** | 1.13 (1.03, 1.24) *** | -0.10 (-0.15, -0.04) *** |
| **Having any IADL disabilities‡** | 0.64 (0.36, 0.92) *** | -0.39 (-0.54, -0.24) *** | 0.93 (0.58, 1.28) *** | -0.22 (-0.40, -0.04) * |
| On preparing a hot meal‡ | 0.81 (0.45, 1.17) *** | -0.34 (-0.53, -0.16) *** | 1.03 (0.48, 1.58) *** | -0.44 (-0.73, -0.16) ** |
| On shopping for groceries‡ | 0.63 (0.33, 0.94) *** | -0.21 (-0.37, -0.05) ** | 1.46 (0.95, 1.97) *** | -0.09 (-0.34, 0.15) |
| On making phone calls‡ | 0.82 (0.38, 1.25) *** | -0.14 (-0.37, 0.08) | 0.82 (0.33, 1.31) *** | -0.24 (-0.48, 0.00) |
| On taking medications‡ | 0.39 (-0.04, 0.81) | -0.38 (-0.59, -0.16) *** | 0.57 (-0.01, 1.15) . | -0.42 (-0.71, -0.13) ** |
| On managing money‡ | 0.61 (0.26, 0.95) *** | -0.65 (-0.83, -0.47) *** | 0.81 (0.34, 1.28) *** | -0.54 (-0.79, -0.30) *** |
| **Social support** | | | | |
| **Number of receipt of any BADL support †** | 0.48 (0.39, 0.57) *** | 0.20 (0.15, 0.24) *** | 0.74 (0.65, 0.83) *** | 0.09 (0.04, 0.13) *** |
| **Receipt of any BADL support‡** | 0.22 (-0.11, 0.55) | -0.01 (-0.18, 0.16) | 0.83 (0.32, 1.34) *** | -0.09 (-0.35, 0.16) |
| On dressing‡ | 0.12 (-0.28, 0.52) | 0.08 (-0.11, 0.28) | 1.00 (0.39, 1.62) *** | -0.09 (-0.39, 0.21) |
| On walking across a room‡ | -0.10 (-0.58, 0.39) | 0.14 (-0.10, 0.38) | 0.74 (-0.25, 1.73) | -0.41 (-0.97, 0.15) |
| On bathing‡ | 0.46 (0.02, 0.89) * | 0.06 (-0.16, 0.28) | 0.81 (-0.03, 1.66) . | -0.25 (-0.72, 0.22) |
| On eating‡ | 0.27 (-0.38, 0.92) | 0.12 (-0.18, 0.42) | 1.00 (-0.05, 2.06) . | 0.05 (-0.47, 0.56) |
| On getting in and out of bed‡ | -0.23 (-0.75, 0.28) | 0.09 (-0.17, 0.34) | 0.96 (0.08, 1.85) * | 0.04 (-0.41, 0.48) |
| On toileting‡ | -0.13 (-0.77, 0.51) | 0.14 (-0.16, 0.44) | 0.42 (-0.86, 1.69) | -0.71 (-1.47, 0.05) |
| **Number of receipt of any IADL support †** | 0.97 (0.88, 1.05) *** | -0.15 (-0.20, -0.11) *** | 0.99 (0.90, 1.08) *** | -0.11 (-0.16, -0.07) *** |
| **Receipt of any IADL support‡** | 0.63 (0.34, 0.92) *** | -0.39 (-0.54, -0.23) *** | 0.94 (0.56, 1.32) *** | -0.28 (-0.47, -0.09) ** |
| On preparing a hot meal‡ | 0.70 (0.30, 1.10) *** | -0.45 (-0.65, -0.24) *** | 0.84 (0.25, 1.43) ** | -0.44 (-0.75, -0.13) ** |
| On shopping for groceries‡ | 0.49 (0.17, 0.80) ** | -0.26 (-0.43, -0.10) ** | 1.39 (0.83, 1.94) *** | -0.17 (-0.44, 0.10) |
| On making phone calls‡ | 0.19 (-0.30, 0.68) | -0.49 (-0.74, -0.23) *** | 0.51 (-0.05, 1.07) | -0.39 (-0.68, -0.11) ** |
| On taking medications‡ | 0.32 (-0.21, 0.84) | -0.41 (-0.69, -0.13) ** | 0.43 (-0.26, 1.11) | -0.47 (-0.81, -0.14) ** |
| On managing money‡ | 0.59 (0.22, 0.97) ** | -0.66 (-0.85, -0.46) *** | 0.76 (0.23, 1.29) ** | -0.69 (-0.98, -0.41) *** |

† Data was fitted by multi-level linear regression model, coefficients represent absolute changes in the outcome with their 95% confidence intervals. ‡ Data was fitted by multi-level logistic regression, coefficients represent log odds of the outcome with their 95% confidence intervals. *** p < 0.001; ** p < 0.01; * p < 0.05.
