# Supplementary material for: Discontinuity of social support among US adults with cognitive impairment before and after the confirmed diagnosis of dementia: a matched ambidirectional cohort study
Source: BMC Med. 2025 Jul 15;23:428. doi: 10.1186/s12916-025-04264-y (PMC12265323; doi:10.1186/s12916-025-04264-y)
Supplement: Supplementary file 4 — Additional file 4: Table S4: Step effect and trend effect of dementia diagnosis on the unmet social support, by overall, matching the control cohort based on all general people [file 12916_2025_4264_MOESM4_ESM.docx]

**Table S4. Step effect and trend effect of dementia diagnosis on the unmet social support, by overall, matching the control cohort based on all general people**.

| **Outcomes** | **Step change** | **Trend change** |
| --- | --- | --- |
| **Number of any unmet BADL support †** | 0.03 (-0.03, 0.08) | -0.02 (-0.04, 0.01) |
| **Having any unmet BADL support ‡** | 0.17 (-0.06, 0.40) | -0.05 (-0.16, 0.07) |
| On dressing ‡ | 0.05 (-0.33, 0.43) | -0.10 (-0.30, 0.09) |
| On walking across a room ‡ | -0.03 (-0.44, 0.37) | 0.03 (-0.17, 0.22) |
| On bathing ‡ | 0.10 (-0.35, 0.54) | -0.03 (-0.26, 0.20) |
| On eating ‡ | -0.40 (-0.98, 0.19) | -0.30 (-0.59, -0.01) * |
| On getting in and out of bed ‡ | -0.04 (-0.45, 0.36) | -0.02 (-0.22, 0.19) |
| On toileting ‡ | 0.35 (-0.04, 0.73) | 0.06 (-0.12, 0.24) |
| **Number of any unmet IADL support †** | 0.10 (0.07, 0.12) *** | 0.02 (0.00, 0.03) ** |
| **Having any unmet IADL support ‡** | 0.21 (-0.11, 0.52) | 0.04 (-0.11, 0.19) |
| On preparing a hot meal ‡ | -0.33 (-0.93, 0.27) | -0.20 (-0.49, 0.09) |
| On shopping for groceries ‡ | 0.85 (0.15, 1.55) * | 0.09 (-0.23, 0.40) |
| On making phone calls ‡ | 0.74 (0.14, 1.34) * | 0.43 (0.15, 0.72) ** |
| On taking medications ‡ | 0.23 (-0.47, 0.93) | -0.23 (-0.63, 0.16) |
| On managing money ‡ | -0.19 (-0.80, 0.42) | -0.10 (-0.40, 0.21) |

† Data was fitted by multi-level linear regression model, coefficients represent absolute changes in the outcome with their 95% confidence intervals. ‡ Data was fitted by multi-level logistic regression, coefficients represent log odds of the outcome with their 95% confidence intervals. *** p < 0.001; ** p < 0.01; * p < 0.05.
