# Supplementary material for: Discontinuity of social support among US adults with cognitive impairment before and after the confirmed diagnosis of dementia: a matched ambidirectional cohort study
Source: BMC Med. 2025 Jul 15;23:428. doi: 10.1186/s12916-025-04264-y (PMC12265323; doi:10.1186/s12916-025-04264-y)
Supplement: Supplementary file 5 — Additional file 5: Table S5: Step effect and trend effect of dementia diagnosis on the unmet social support, by race/ethnicity, matching the control cohort based on all general people [file 12916_2025_4264_MOESM5_ESM.docx]

**Table S5. Step effect and trend effect of dementia diagnosis on the unmet social support, by race/ethnicity, matching the control cohort based on all general people**.

| **Outcomes** | **Hispanic** | | **Non-Hispanic Black** | | **Non-Hispanic White** | |
| --- | --- | --- | --- | --- | --- | --- |
|  | Step change | Trend change | Step change | Trend change | Step change | Trend change |
| **Number of any unmet BADL support** **†** | 0.13 (-0.09, 0.35) | 0.01 (-0.09, 0.12) | 0.20 (0.02, 0.38) * | -0.07 (-0.16, 0.02) | -0.02 (-0.08, 0.04) | -0.01 (-0.04, 0.02) |
| **Having any unmet BADL support** **‡** | 0.80 (0.08, 1.52) * | 0.05 (-0.29, 0.38) | 0.64 (0.05, 1.22) * | -0.05 (-0.33, 0.22) | -0.02 (-0.29, 0.26) | -0.05 (-0.19, 0.08) |
| On dressing**‡** | 0.31 (-0.74, 1.36) | 0.21 (-0.29, 0.71) | 0.41 (-0.53, 1.34) | -0.09 (-0.57, 0.39) | -0.11 (-0.56, 0.34) | -0.18 (-0.42, 0.06) |
| On walking across a room**‡** | 1.11 (-0.29, 2.50) | -0.15 (-0.80, 0.49) | 0.46 (-0.51, 1.43) | -0.39 (-0.86, 0.08) | -0.26 (-0.73, 0.22) | 0.15 (-0.08, 0.38) |
| On bathing**‡** | -0.33 (-1.68, 1.02) | 0.48 (-0.15, 1.11) | -0.08 (-1.19, 1.04) | -0.26 (-0.83, 0.30) | 0.22 (-0.31, 0.75) | -0.04 (-0.32, 0.23) |
| On eating**‡** | 0.22 (-1.26, 1.69) | -0.26 (-0.89, 0.37) | -0.39 (-2.13, 1.35) | -0.57 (-1.50, 0.37) | -0.61 (-1.32, 0.10) | -0.30 (-0.67, 0.07) |
| On getting in and out of bed**‡** | -0.28 (-1.26, 0.70) | -0.30 (-0.78, 0.19) | -0.32 (-1.28, 0.64) | -0.14 (-0.62, 0.34) | 0.06 (-0.44, 0.56) | 0.14 (-0.12, 0.40) |
| On toileting**‡** | 0.83 (-0.59, 2.25) | 0.34 (-0.29, 0.97) | 1.55 (0.58, 2.52) ** | 0.05 (-0.39, 0.48) | 0.00 (-0.44, 0.45) | 0.07 (-0.15, 0.28) |
| **Number of any unmet IADL support** **†** | 0.10 (0.02, 0.19) * | -0.01 (-0.05, 0.04) | 0.07 (-0.01, 0.14) | 0.00 (-0.03, 0.04) | 0.10 (0.07, 0.13) *** | 0.03 (0.01, 0.04) *** |
| **Having any unmet IADL support‡** | 0.68 (-0.36, 1.72) | 0.14 (-0.36, 0.64) | 0.14 (-0.66, 0.95) | 0.26 (-0.12, 0.65) | 0.16 (-0.21, 0.53) | -0.00 (-0.18, 0.18) |
| On preparing a hot meal**‡** | -2.65 (-4.99, -0.32) * | -0.79 (-1.97, 0.39) | -0.43 (-1.95, 1.09) | -0.15 (-0.93, 0.62) | -0.11 (-0.79, 0.58) | -0.16 (-0.49, 0.16) |
| On shopping for groceries**‡** | 2.24 (-0.02, 4.49) | 1.12 (0.11, 2.13) * | 0.96 (-1.02, 2.93) | 0.66 (-0.20, 1.51) | 0.62 (-0.18, 1.42) | -0.12 (-0.48, 0.25) |
| On making phone calls**‡** | 0.53 (-1.40, 2.46) | 0.67 (-0.19, 1.52) | -0.60 (-2.45, 1.25) | 0.24 (-0.61, 1.08) | 0.89 (0.19, 1.59) * | 0.41 (0.07, 0.75) * |
| On taking medications**‡** | 2.46 (0.43, 4.49) * | -0.22 (-1.23, 0.78) | 0.65 (-0.83, 2.13) | -0.59 (-1.57, 0.38) | -0.53 (-1.43, 0.37) | -0.03 (-0.51, 0.46) |
| On managing money**‡** | -0.30 (-2.20, 1.60) | -0.14 (-1.09, 0.80) | 0.17 (-1.35, 1.68) | -0.00 (-0.71, 0.71) | -0.22 (-0.94, 0.50) | -0.09 (-0.46, 0.28) |

† Data was fitted by multi-level linear regression model, coefficients represent absolute changes in the outcome with their 95% confidence intervals. ‡ Data was fitted by multi-level logistic regression, coefficients represent log odds of the outcome with their 95% confidence intervals. *** p < 0.001; ** p < 0.01; * p < 0.05..
