# Supplementary material for: Discontinuity of social support among US adults with cognitive impairment before and after the confirmed diagnosis of dementia: a matched ambidirectional cohort study
Source: BMC Med. 2025 Jul 15;23:428. doi: 10.1186/s12916-025-04264-y (PMC12265323; doi:10.1186/s12916-025-04264-y)
Supplement: Supplementary file 6 — Additional file 6: Table S6: Step effect and trend effect of dementia diagnosis on the unmet social support, by sex, matching the control cohort based on all general people [file 12916_2025_4264_MOESM6_ESM.docx]

**Table S6. Step effect and trend effect of dementia diagnosis on the unmet social support, by sex, matching the control cohort based on all general people**.

| **Outcomes** | **Female** | | **Male** | |
| --- | --- | --- | --- | --- |
|  | Step change | Trend change | Step change | Trend change |
| **Number of any unmet BADL support†** | 0.01 (-0.07, 0.08) | -0.01 (-0.04, 0.03) | 0.08 (-0.00, 0.16) | -0.03 (-0.07, 0.01) |
| **Having any unmet BADL support‡** | 0.16 (-0.13, 0.45) | 0.00 (-0.14, 0.15) | 0.19 (-0.20, 0.57) | -0.12 (-0.31, 0.07) |
| On dressing**‡** | 0.25 (-0.26, 0.76) | -0.06 (-0.32, 0.20) | -0.19 (-0.76, 0.37) | -0.15 (-0.44, 0.15) |
| On walking across a room**‡** | -0.11 (-0.62, 0.40) | 0.07 (-0.17, 0.32) | 0.11 (-0.56, 0.79) | -0.01 (-0.35, 0.33) |
| On bathing**‡** | 0.17 (-0.36, 0.71) | -0.03 (-0.31, 0.24) | -0.16 (-0.98, 0.66) | -0.06 (-0.47, 0.35) |
| On eating**‡** | -0.44 (-1.19, 0.31) | -0.28 (-0.64, 0.08) | -0.21 (-1.18, 0.77) | -0.21 (-0.72, 0.30) |
| On getting in and out of bed**‡** | -0.24 (-0.75, 0.27) | -0.08 (-0.33, 0.18) | 0.37 (-0.30, 1.04) | 0.13 (-0.21, 0.46) |
| On toileting**‡** | 0.11 (-0.34, 0.56) | 0.08 (-0.13, 0.30) | 0.89 (0.10, 1.68) * | 0.05 (-0.30, 0.41) |
| **Number of any unmet IADL support†** | 0.07 (0.03, 0.10) *** | 0.01 (-0.00, 0.03) | 0.14 (0.10, 0.17) *** | 0.02 (0.01, 0.04) ** |
| **Having any unmet IADL support‡** | -0.00 (-0.39, 0.39) | 0.05 (-0.14, 0.24) | 0.58 (0.04, 1.12) * | 0.01 (-0.25, 0.27) |
| On preparing a hot meal**‡** | -0.66 (-1.35, 0.03) | -0.22 (-0.56, 0.11) | 0.45 (-0.84, 1.74) | -0.27 (-0.92, 0.38) |
| On shopping for groceries**‡** | 0.88 (0.01, 1.76) * | 0.08 (-0.32, 0.48) | 0.56 (-0.67, 1.79) | -0.16 (-0.71, 0.40) |
| On making phone calls**‡** | 0.76 (-0.03, 1.56) | 0.54 (0.16, 0.92) ** | 0.73 (-0.19, 1.65) | 0.34 (-0.10, 0.78) |
| On taking medications**‡** | -0.23 (-1.08, 0.62) | -0.21 (-0.68, 0.26) | 1.35 (0.07, 2.63) * | -0.30 (-1.01, 0.41) |
| On managing money**‡** | -0.33 (-1.08, 0.41) | -0.29 (-0.68, 0.09) | 0.15 (-0.90, 1.20) | 0.26 (-0.25, 0.76) |

† Data was fitted by multi-level linear regression model, coefficients represent absolute changes in the outcome with their 95% confidence intervals. ‡ Data was fitted by multi-level logistic regression, coefficients represent log odds of the outcome with their 95% confidence intervals. *** p < 0.001; ** p < 0.01; * p < 0.05.
