# Supplementary material for: Discontinuity of social support among US adults with cognitive impairment before and after the confirmed diagnosis of dementia: a matched ambidirectional cohort study
Source: BMC Med. 2025 Jul 15;23:428. doi: 10.1186/s12916-025-04264-y (PMC12265323; doi:10.1186/s12916-025-04264-y)

**Figure S3. Controlled interrupted time series analysis of unmet social support in dementia diagnosis cohort (square points) and non-diagnosis cohort (triangle points), by sex, matching the control cohort based on all general people.** The dashed or solid lines are the model fit for two cohorts.


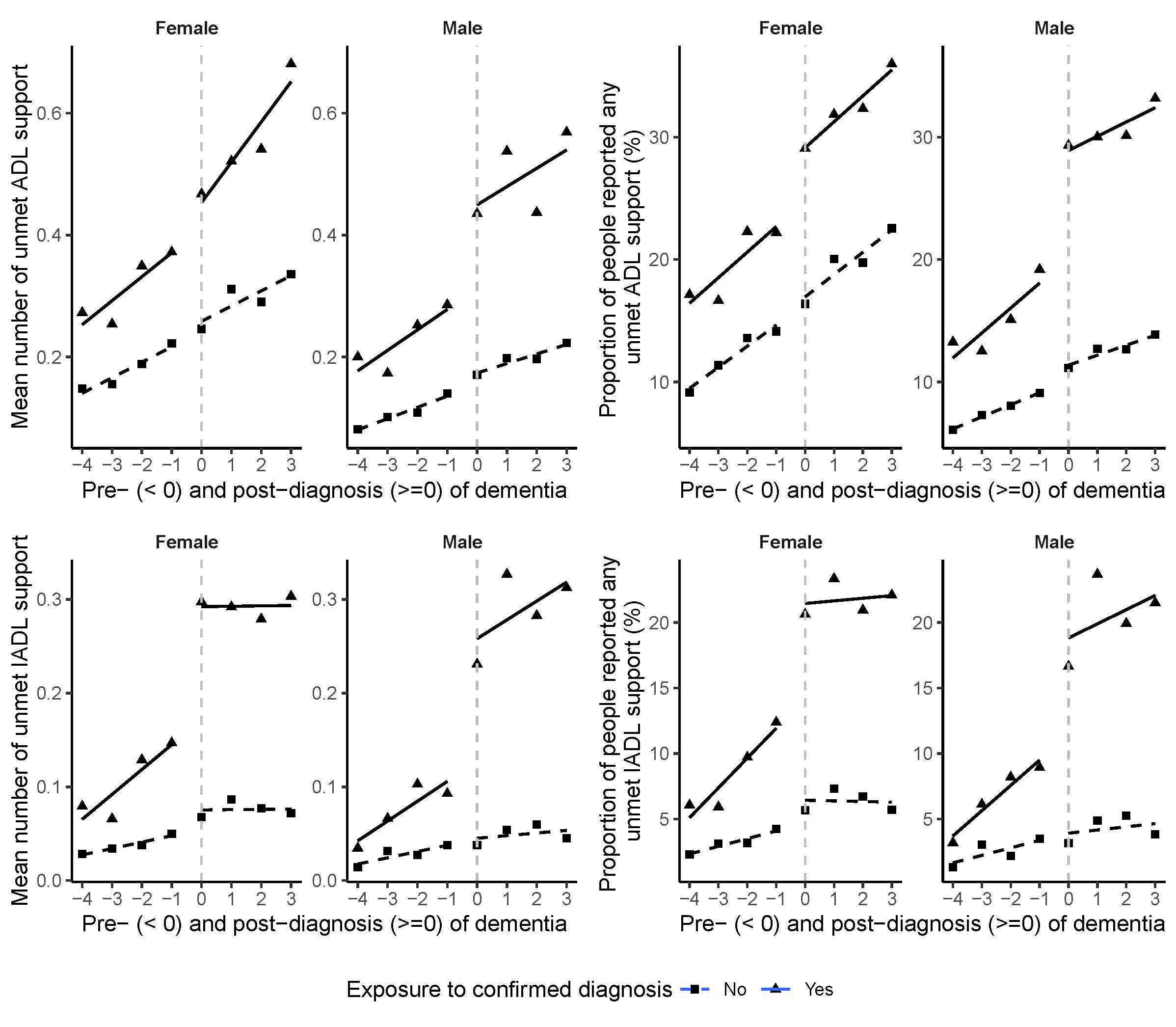

Supplement: Supplementary file 9 — Additional file 9: Figure S3: Controlled interrupted time series analysis of unmet social support in dementia diagnosis cohortand non-diagnosis cohort, by sex, matching the control cohort based on all general people [file 12916_2025_4264_MOESM9_ESM.docx]
